# Effect of transferrin glycation induced by high glucose on HK-2 cells *in vitro*

**DOI:** 10.3389/fendo.2022.1009507

**Published:** 2023-01-26

**Authors:** Yanqi Ma, Qikai Zhou, Pingping Zhao, Xiaoyu Lv, Caixia Gong, Jie Gao, Jingfang Liu

**Affiliations:** ^1^ The First Clinical Medical College, Lanzhou University, Lanzhou, Gansu, China; ^2^ Tianjin Normal University, Tianjin, China; ^3^ Department of Endocrinology, The First Hospital of Lanzhou University, Lanzhou, Gansu, China

**Keywords:** glycation, transferrin, advanced glycation end modified transferrin, HK-2, transferrin receptor

## Abstract

**Background and objective:**

Glycation is a common post-transcriptional modification of proteins. Previous studies have shown that advanced glycation end modified transferrin (AGE-Tf) levels in diabetic rat kidney tissues were increased; however, its role in diabetic nephropathy remains unclear. In this study, differences in glycation degree and Tf sites induced by differing high glucose concentrations *in vitro* and the effect on total iron binding capacity (TIBC) were observed. Moreover, the effect of AGE-Tf on human renal tubular epithelial cells (HK-2) was investigated.

**Methods:**

*In vitro* Tf was incubated with increasing glucose concentrations (0 mM, 5.6 mM, 11.1 mM, 33.3 mM, 100 mM, 500 mM, and 1,000 mM) for AGE-Tf. Differences in AGE-Tf glycation degree and TIBC level were analyzed *via* colorimetric method. The AGE-Tf glycation sites were identified with LC-MS/MS. HK-2 cells were treated with AGE-Tf prepared with different glucose concentrations (33.3 mM and 500 mM) *in vitro*. The effects of AGE-Tf on HK-2 cell viability, proliferation, oxidative stress index, and Tf receptor expression levels were then observed.

**Results:**

With increasing glucose concentrations (100 mM, 500 mM, and 1,000 mM) *in vitro*, Tf glycation degree was significantly increased. The TIBC levels of AGE-Tf were decreased significantly with increasing glucose concentrations (33.3 mM, 100 mM, 500 mM, and 1,000 mM). Four glycated modification sites in Tf and 17 glycated modification sites were detected in AGE-Tf (500 mM) by LC-MS/MS. The structural types of AGEs were CML, G-H1, FL-1H2O, FL, and MG-H1. No significant differences were found in the survival rate of HK-2 cells among the AGE-Tf (500 mM), AGE-Tf (33.3 mM), and Tf groups (all *p* > 0.05). The apoptosis rate of HK-2 cells in the AGE-Tf (500 mM) group was significantly higher than that in the AGE-Tf (33.3 mM) group. Additionally, both of them were significantly higher than that in the Tf group (both *p* < 0.05). The MDA levels of HK-2 cells in the AGE-Tf (500 mM) and AGE-Tf (33.3 mM) groups were higher than that in the Tf group, but not significantly (both *p* > 0.05). The T-AOC level of HK-2 in the AGE-Tf (500 mM) group was significantly lower than that in the AGE-Tf (33.3 mM) and Tf groups (both *p* < 0.001). The GSH level of HK-2 cells in the AGE-Tf (500 mM) group was significantly lower than that in the Tf group (*p* < 0.05). The expression level of TfR in the AGE-Tf (500 mM) group was also significantly lower than that in the Tf group (*p* < 0.05).

**Conclusion:**

The degree and sites of Tf glycation were increased *in vitro* secondary to high-glucose exposure; however, the binding ability of Tf to iron decreased gradually. After HK-2 was stimulated by AGE-Tf *in vitro*, the apoptosis of cells was increased, antioxidant capacity was decreased, and TfR expression levels were downregulated.

## 1 Introduction

Glycation refers to a common post-transcriptional modification of proteins. Non-enzymatic glycation, also known as “Maillard” reaction, is a spontaneous, cascading, non-enzyme-catalyzed, complex, site-specific reaction. It is the condensation of aldehyde or ketone groups of reducing sugars (glucose, fructose, and galactose) with ϵ-amino groups of lysine or hydroxylysine in proteins to form Schiff bases. Schiff bases undergo a series of cyclization, dehydration, and rearrangement to form various compounds called advanced glycation end products (AGEs) (1, 2). Currently, many AGE-modified proteins, such as hemoglobin, albumin, collagen, lens protein, and erythrocyte membrane proteins, have been reported (1, 3–5).

Diabetes is a metabolic disease characterized by hyperglycemia. Its complications, including nephropathy, retinopathy, atherosclerosis, and neuropathy, severely threaten human health (6). Hyperglycemia is the main factor affecting the occurrence and development of diabetic complications. The glycation hypothesis suggests that high glucose may accelerate non-enzymatic glycation of proteins. It affects the structure, function, and turnover of proteins, as well as the gradual decline of tissue function (7). Recently, with the continuous improvements in technologies, such as proteomics and glycometabolomics, and the rapid development of related detection technologies, including high-performance liquid chromatography/electrospray ionization-mass spectrometry (HPLC/ESI-MS), studies have shown that non-enzymatic glycated modification of transferrin (Tf) is closely related to the occurrence and development of diabetes and its complications (8–12).

Compared to non-diabetic patients, the degree of Tf glycation in the serum of diabetic patients has increased (13). In addition, not only the serum levels of glycated Tf (14), but also the kidney tissue levels of glycated Tf in diabetic rats have increased (15). Glycation damages the iron-binding capacity of Tf (11), contributing to increased oxidative stress and occurrence of non-Tf-bound iron species (NTBI) (16). The prevalence of NTBI in diabetes has been described (12). *In vitro*, under high sugar state, Lys206 and Lys534 in apo-Tf (9) or Lys103, Lys312, and Lys382 in holo-Tf (10) are most sensitive to glycation. The specific sites of glycation may affect the normal structure and function of Tf. However, little is known about the specificity of these glycation sites. Molecular dynamic simulations also suggested that additional loss of iron binding capacity may result from the stereochemical effects induced by the glycation of lysine residues. These prevent the conformational changes required for metal binding (17). In addition to affecting iron transport, Tf glycation may also hinder transferrin receptor (TfR) binding (10).

AGE formation is accompanied by reactive oxygen species (ROS) production, which further damages proteins and other important biological molecules (18). Sustained exposure to AGEs causes HK-2 cells to undergo epithelial-to-mesenchymal transition (EMT). HK-2 cells treated with AGE-modified bovine serum albumin (AGE-BSA) promoted the expression of EMT-related proteins, while decreasing the expression of the epithelial cell marker E-cadherin (19). AGE-BSA stimulates protein kinase C (PKC) activity, increases transforming growth factor-beta (TGF-beta) bio-activity, induces gene overexpression, and enhances the production of extracellular matrix proteins in glomerular mesangial and endothelial cells (20).

Until now, the effect of Tf glycation on HK-2 remains unclear. In this study, differences in glycation degree and Tf sites induced by different high glucose concentrations *in vitro* as well as the effect on its total iron binding capacity (TIBC) were observed. Moreover, the effects of AGE-Tf on HK-2 cell viability, proliferation, oxidative stress index, and Tf receptor expression level were investigated to provide novel avenues for diagnosing and treating diabetic nephropathy.

## 2 Materials and methods

### 2.1 Materials and reagents

Human serum APO-Tf (T1147) was obtained from Sigma. The TfR primary antibody (13208s) was purchased from the American CST Company. The β-actin antibody (20536-1-AP) was purchased from Proteintech Group, Inc. HK-2 (Procell CL-0109) human proximal renal tubular epithelial cells (TECs) were provided by Wuhan Punosai Life Science and Technology Co., Ltd. Acetonitrile (Fisher Scientific, A955-4) and chymotrypsin (Promega, V5111) at mass spectrometry level were purchased.

### 2.2 Preparation of glycated Tf

Glycated Tf was obtained by incubating human serum apo-Tf (5 mg/ml) in sodium phosphate buffer (pH 7.4, 0.1 mM), with various concentrations of D-glucose (0 mM, 5.6 mM, 11.1 mM, 33.3 mM, 100 mM, 500 mM, and 1,000 mM). These were filtered and sterilized using a needle filter with a diameter of 0.22 μm and then encapsulated and incubated at 37°C for 14 days. Excess glucose was removed by cycles of concentration and dilution in sodium phosphate buffer. Aliquots of the different samples were stored at −80°C.

### 2.3 Identification of glycated sites in AGE-Tf and Tf by LC-MS/MS

Ten microliters of protein samples was taken and 90 µl of 2×DOC lysis buffer was added. Then, we added dithiothreitol (DTT) (Promega Corporation) to a final concentration of 10 mM, heated to 100°C for 5 min, and cooled to room temperature. We next added 8 µl of 500 mM iodoacetamide (IAA) [Promega Corporation] (final concentration, 40 mM), mixed it well, and allowed it to react in the dark for 1 h. The protein lysate was then transferred to a 10-kDa filter and centrifuged at 14,000 *g* for 10 min, and 300 µl of 50 mM NH_4_HCO_3_ was added. The solution was mixed five times with a pipette and centrifuged at 14,000 *g* for 10 min. The filtrate was poured off, 50 µl of NH_4_HCO_3_ was added, and it was centrifuged again at 14,000 *g* for 5 min. The filtrate was poured and the bottom of the tube was washed twice with 500 µl of mass spectrometry-grade water (the volume of water is greater than the volume of the filtrate). Fifty microliters of 50 mM NH_4_HCO_3_ and 0.5 µl of chymotrypsin were added and mixed thoroughly. The tube was then covered with parafilm and digested at 37°C for 4 h. Next, 0.5 µg of chymotrypsin was added, mixed well, and sealed with parafilm. After overnight incubation at 37°C, the filter was taken out, and centrifuged at 14,000 *g* for 10 min. The digested peptides were then collected and washed twice with 50 mM NH_4_HCO_3_.

Twenty microliters of 10% FA was added to the peptide and then DOC precipitation was done. The sample was centrifuged at 14,000 *g* for 10 min at room temperature, and the supernatant was taken for desalting. Eighty microliters of 100% ACN was added twice to activate the column and centrifuged at 2,000 *g* for 1 min. Afterwards, 80 µl of 0.1% FA was added twice to equilibrate the column then centrifuged at 3,300 *g* for 1 min. Again, 80 µl of sample was centrifuged at 3,300 *g* for 1 min and washed twice with 80 µl of 0.1% FA, then centrifuged at 3,300 *g* for 1 min. Finally, 80 µl of 50% ACN + 0.1% FA was used to elute the sample once. The sample was then dried for 1 h at 1,800 rpm, and stored at −80°C.

The liquid chromatography system used was Easy-nLC1200. The sample was dissolved in 140 µl of mobile phase A (0.1% formic acid aqueous solution), shaken, and mixed evenly, and then 1 µl of sample was added to a 20-µl loop. Pump A loaded the sample in the loop to a C18 reverse-phase precolumn. A pump loaded the sample in the quantitative ring onto the C18 reverse-phase precolumn at a maximum pressure of 280 bar. The sample was then eluted onto the C18 analytical column for separation using 11%–95% mobile phase B (0.1% formic acid+80% acetonitrile) at a flow rate of 600 nl/min.

An Orbitrap Fusion Lumos mass spectrometer was used for data collection. The ion source was a nano-current electrospray ion source (NSI), the spray voltage was 2,200 V, and the ion transfer capillary temperature was 320°C. Mass spectrometry data were collected using data-dependent acquisition (DDA) in positive ion mode. Orbitrap was used for full scanning at level 1. The scanning range was *m*/*z* 300–2,000, scan resolution was 120,000, automatic gain control (AGC) was 4 × 10^5^ ions, and max injection time was 50 ms. Furthermore, mass spectrometry was used to fragment the parent ions by 40% high-energy collision dissociation (HCD). The fragments were detected in Orbitrap with a first mass of 100, a resolution of 30,000, and AGC targets of 5 × 10^4^ ions. Max injection time was 54 ms. The parent ion selection window was set as 1.6, and MS/MS collection was carried out for ions with two to seven charge numbers. The dynamic exclusion was set so that the same parent ion would not be collected by MS/MS within 30 s after each parent ion was collected by MS/MS for five times.

### 2.4 Determination of the degree of Tf glycation and TIBC under different glucose states *in vitro*

According to the fructosamine kit (ab228558) instructions, all the ingredients and prepared reagent were rewarmed to room temperature and stirred gently before the experiment. Next, 10 µl of undiluted sample or 10 μl of dH_2_O was added to a clean 96-well plate and labeled as experimental or blank group. Three microliters of fructose amine standard was then added as the positive control group, supplemented with 10 μl of buffer A followed by 37 μl of mix (2 µl of recombinant mercaptan blocker + 5 µl of recombinant sample cleaning mixture + 30 µl of A buffer) to each well. Thirty microliters of NBT was next added to each tube and incubated at 37°C for 10 min under dark conditions. Afterwards, 200 μl of B buffer was added to the tubes successively. The absorbance of each well was measured at 530 nm wavelength at 37°C with a microplate reader. The fructoamine content of each group was then calculated.

According to the TIBC kit (Nanjing Jiancheng, China, A040-1-1) instructions, the absorbance of each tube (OD value) was measured. The TIBC in each tube sample was calculated by the following formula: TIBC (mg/L) = (determination OD value − blank OD value)/(standard OD value − blank OD value) × standard concentration (1 mg/L) × dilution ratio before test.

### 2.5 HK-2 cells culture

HK-2 cells were cultured in MEM medium containing 10% FBS (BI, 04-001-1ACS) in an incubator (37°C, 5% CO_2_). When the cells reached about 80%–90% fusion, trypsin (BI, 03-050-1BCS) was used for digestion, and the collected cells were re-suspended in complete culture medium to make single-cell suspensions for subsequent experiments.

#### 2.5.1 Experimental group

The experiment was divided into a blank (NC) group (MEM medium without any stimulant), a negative control (Tf) group (non-glycated Tf), an AGE-Tf (33.3 mM) group (33.3 mM glucose in glycated Tf), and an AGE-Tf (500 mM) group (500 mM glucose in glycated Tf).

#### 2.5.2 Cell viability assay

HK-2 cells were inoculated into 96-well plates at 1 × 10^3^/well, and different stimuli were given according to experimental groups. Then, 10 μl of CCK-8 solution (APEXBIO, K1018) was added to each well and the culture plate was gently tapped to completely mix the reagent. Then, the absorbance was measured at 450 nm after incubation for 2 h without light, and the experimental results were recorded and analyzed with GraphPad Prism 5 software.

#### 2.5.3 Cell apoptosis detection

HK-2 cells were inoculated into six-well plates at 1 × 10^5^/well, and different stimuli were given according to the groups. Based on the instructions of the apoptosis detection kit (Beibo, China), cell supernatant was collected and transferred to a 15-ml tube. This was centrifuged at 300 g and 4°C for 5 min when the intervention time was reached. Then, the supernatant was discarded and cell precipitation was reserved. Adherent cells were digested with trypsin without EDTA and centrifuged at 300–500 *g* for 5 min in 4°C. These were washed using cold PBS twice, with centrifugation each time. The supernatant was discarded and the sample was collected along with cells from the previous medium. The cells were suspended in 400 µl of 1× Annexin V binding solution at a concentration of about 1 × 10^6^ cells/ml, mixed gently, and incubated at 4°C for 15 min under dark conditions when 5 µl of Annexin V-FITC staining solution was added to the cell suspension. After adding 5 µl of 7-AAD staining solution, the cells were gently mixed, incubated for 5 min at 4°C under dark conditions, and detected by flow cytometry.

#### 2.5.4 Determination of oxidation and antioxidant indexes

HK-2 cells were inoculated into six-well plates at 1 × 10^5^/well. The cells were then digested with trypsin and collected. MDA (Nanjing Jiancheng, China, A003-2-2), T-AOC (Solarbio, Beijing, China, BC1310), and GSH (Nanjing Jiancheng, China, A006-2-1) levels were detected according to the instructions.

#### 2.5.5 RNA extraction and RT-PCR

The cell/tissue total RNA extraction kit (Shanghai YE SEN, China) was used to obtain the total RNA of cells and synthesized into cDNA by reverse transcription (TaKaRa, Beijing, China). After the original solution was diluted five times, 2× ChamQ SYBR Qpcr Master Mix (Vazyme, China) was used for the RT-PCR reaction. Each group was set with three multiple wells. The primer sequences were as follows: TfR primer sequence—upstream 5’-TTCCACCATCTCGGTCATCA-3’, downstream 5’-AGGTATCCCTCTAGCCATTCAGT-3’; GAPDH primer sequence—upstream 5’-GGAGCGAGATCCCTCCAAAAT-3’, downstream 5’-GGCTGTTGTCATACTTCTCATGG-3’. The 2^−ΔΔCt^ method was used to calculate the relative expression of mRNA.

#### 2.5.6 Western blot analysis

After the cells were lysed using RIPA lysis solution (Solarbio, Beijing, China, R0010), samples were centrifuged at 12,000 rpm for 20 min at 4°C to collect the supernatant and obtain protein samples. Subsequently, the protein concentration was measured using the BCA kit (Beyotime Biotechnology, Shanghai, China). Then, the corresponding volume of proteins was mixed with the loading buffer (Beyotime Biotechnology, Shanghai, China) and boiled in water for 10 min to denature the proteins. Subsequently, the protein samples were separated by SDS-PAGE and transferred into PVDF membranes. Next, the membranes were placed in the blocking solution containing 2% BSA for 1 h at indoor temperature. Afterwards, primary antibodies were added, including rabbit anti-human TfR (diluted 1:1,000) and anti-β-actin antibody (diluted 1:500). Antibodies were kept at 4°C overnight. The membrane was washed with TBST thrice for 10 min each. The membranes were then transferred into the secondary antibody (horseradish peroxidase-labeled goat anti-rabbit IgG, 1:1,000; Boster Biological Technology Co. Ltd.) and incubated for 1 h at room temperature. Then, the membranes were washed for 10 min thrice again. After the developer is dropped onto the membrane, detection is performed using a chemiluminescence imaging system (USA Protein Simple).

### 2.6 Statistical analysis

The experimental data obtained in this study were analyzed by SPSS 22.0 software. The results were expressed as mean ± standard deviation (*x* ± s). *t*-test was used to compare two groups, and one-way ANOVA was used for multiple groups. All graphs in this study were made by GraphPad Prism 8.4.3 software, and *p* < 0.05 was considered as statistically significant.

## 3 Results

### 3.1 Comparison of Tf glycation degree and TIBC under different glucose states *in vitro*

**Figure 1:**
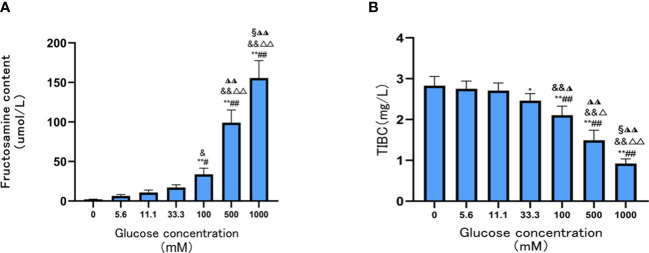
Contents of Tf fructosamine and TIBC level prepared under different glucose concentrations. **(A)** The contents of Tf fructosamine prepared under different glucose concentrations; **(B)** TIBC levels of Tf prepared at different glucose concentrations. Compared with 0mM group, *P < 0.05, **P < 0.001;Compared with the 5.6mM group, #P < 0.05, ##P < 0.001;Compared with the 11.1mM group, &P < 0.05, && P < 0.001; Compared with the 33.3mM group, ◮P < 0.05, ◮◮P < 0.001; Compared with the 100mM group, △P < 0.05, △△P < 0.001; Compared with the 500mM group, §P < 0.05.

Compared with the Tf group, fructosamine contents were significantly increased when glucose concentration was 100 mM, 500 mM, and 1,000 mM (all *p* < 0.05). This suggests that glucose concentration is directly associated with the degree of Tf glycation; the degree of Tf glycation was highest when the glucose concentration was 1,000 mM (all *p* < 0.05) (**Figure 1A**).

The TIBC levels of AGE-Tf were significantly decreased with increasing glucose concentrations(33.3 mM, 100 mM, 500 mM, and 1,000 mM); the reduction was most significant when the glucose concentration was 1,000 mM (*p* < 0.05) (**Figure 1B**).

### 3.2 Identification of Tf glycation sites by LC-MS/MS

**Figure 2:**
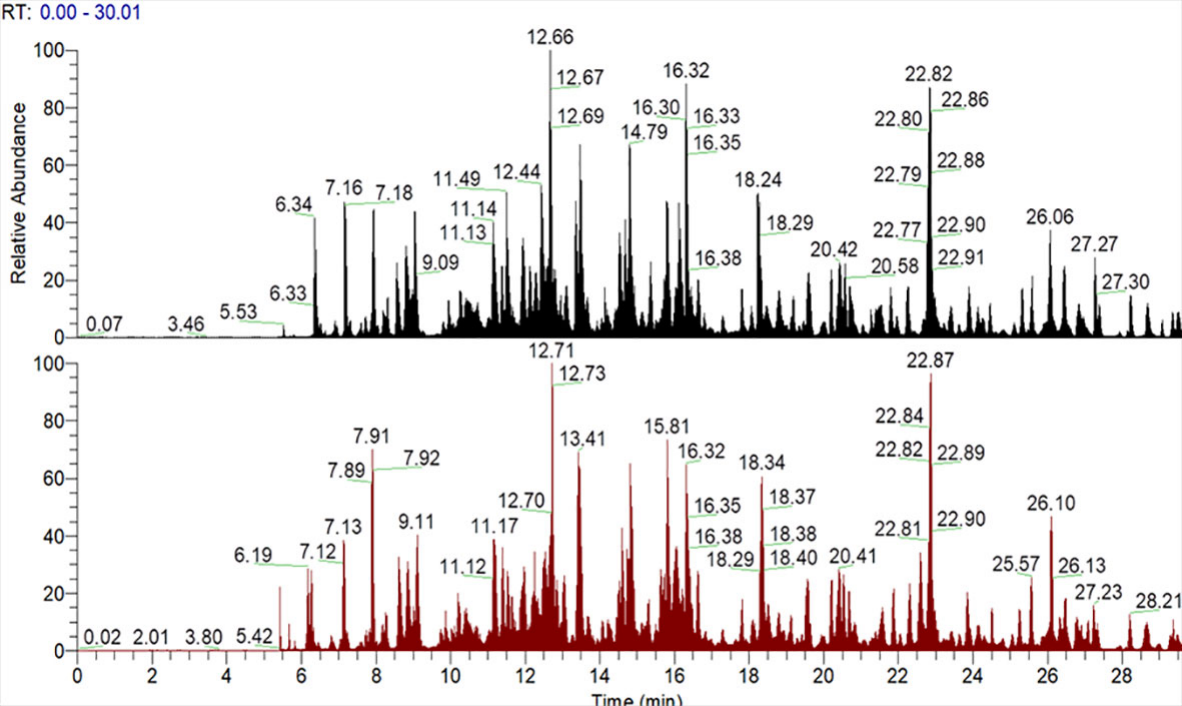
Mass spectrometry signal total ion flow chromatogram. Note: the abscissa is the elution time and the ordinate is the peak intensity. The figure above is Tf; the following figure shows the AGE-Tf (500 mM) sample, that is, AGE-Tf with 500 mM glucose.

The glycation sites of Tf and AGE-Tf (500 mM) were detected with LC-MS/MS technology. After completing the MS scan, the total ion flow chromatogram of MS signal was obtained. The abscissa marked the elution time and ordinate for peak intensity (**Figure 2**).

Four glycation modification sites (R69, K384, K399, and R587) were detected in Tf, and the glycation sites of AGE-Tf (500 mM) increased to 17 (R69, K121, K225, K236, K258, K297, R346, K359, K362, K384, K399, K546, K553, K564, R587, K659, and K676). The main structural types of AGE were CML, G-H1, FL-1H2O, FL, and MG-H1 (**Table 1**).

**Table 1:** Identification of Tf glycation sites by LC-MS/MS.

Type	Sequence	Site	AGEs type	Abundance	*m*/*z* [Da]	DeltaM [ppm]	RT [min]	XCorr
**Tf**	DCIR*AIAANEADAVTLDAGL	R69	CML	0.04785165	774.7144	0.21	24.711	4.84
	SVNSVGK*IECVSAETTEDCIAKIMNGEADAMSLDGGFVY	K384	CML	0.04215376	1,395.963	6.06	28.2125	3.1
	SVNSVGKIECVSAETTEDCIAK*IMNGEADAMSLDGGFVY	K399	CML	0.44459433	1,046.973	0.92	24.6166	5.73
	CLDGTR*KPVEEYANCHL	R587	G-H1	0.20028566	682.3123	0.44	17.2652	6.15
AGE-Tf (500)	DCIR*AIAANEADAVTLDAGL	R69	CML	0.01471251	774.7146	0.45	24.7008	4.38
	AVAVVK*KDSGF	K121	FL-1H2O	0.00301770	632.8427	−0.28	8.7409	1.4
	KDGAGDVAFVK*HSTIFENL	K225	FL	0.40914975	737.3713	0.79	19.4506	4.29
	ENLANK*ADRDQY	K236	FL	0.02814940	799.8686	0.41	7.0587	2.63
	CLDNTRKPVDEYK*DCHL	K258	FL	0.00320289	775.6875	−0.26	10.0912	2.9
	NQAQEHFGKDK*SKEF	K297	FL	0.00174538	1,058.988	0.9	6.1656	2.76
	EYVTAIRNLR*EGTCPEAPTD	R346	CML	0.00539881	805.8928	2.81	16.8859	5.64
	REGTCPEAPTDECK*PVKW	K359	FL	0.05737817	774.684	−0.14	11.0157	4.42
	VTAIRNLREGTCPEAPTDECKPVK*W	K362	FL	0.00212094	1,084.523	−0.02	13.3267	4.39
	SVNSVGK*IECVSAETTEDCIAKIMNGEADAMSLDGGFVY	K384	CML	0.11824112	1,390.62646	2.47	27.8645	3.94
	SVNSVGKIECVSAETTEDCIAK*IMNGEADAMSLDGGFVY	K399	CML	0.29365557	1,057.23206	3.41	24.2108	6.92
	RCLVEK*GDVAFVKHQTVPQNTGGKNPDPW	K546	MG-H1	0.00105743	688.7466	5.51	13.8979	4.66
	VEKGDVAFVK*HQTVPQNTGGKNPDPW	K553	MG-H1	0.10691040	753.3778	−0.43	12.8532	4.65
	RCLVEKGDVAFVKHQTVPQNTGGK*NPDPW	K564	MG-H1	0.00105743	688.7466	5.51	13.8979	4.66
	CLDGTR*KPVEEYANCHL	R587	G-H1	0.04885650	682.3125	0.7	17.2432	6.04
	RDDTVCLAK*LHDRNTY	K659	FL	0.00961104	713.6762	0.19	10.6869	4.27
	LGEEYVK*AVGNL	K676	FL	0.01821182	727.3749	0.3	19.2628	2.5

Tf: transferring; AGE-Tf (500 mM): prepared under the condition of glucose concentration of 500 mM. K* indicates that the modified site is lysine. R* indicates that the modified site is arginine. CML: N epsilon-carboxymethyl-lysine; FL: Fructosyl lysine; H2o FL-1: Fructosyl lysine-1 h2o; MG-H1: N epsilon-(5-veidekke-5-methyl-4-imidazolon-2-yl) ornithine (MG-H1).

### 3.3 Effect of AGE-Tf on HK-2 cell viability

**Figure 3:**
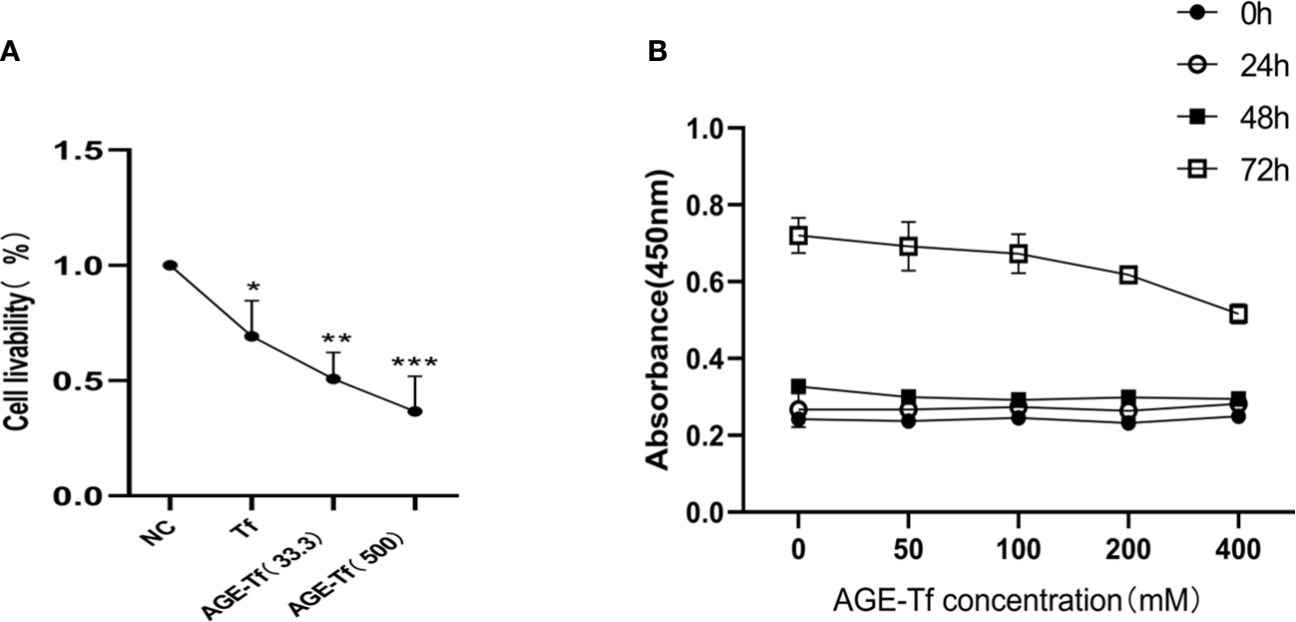
Effect of AGE-Tf on HK-2 cell viability. **(A)** Effect of Tf glycation on survival rate of HK-2. **(B)** Survival of HK-2 cells under different AGE-Tf concentrations and time points. Compared with the NC group, *p < 0.05, **p < 0.01, ***p < 0.001; AGE-Tf (33.3): the glucose concentration was 33.3 mM when preparing AGE-Tf; AGE-Tf (500): the glucose concentration was 500 mM when preparing AGE-Tf.

Compared with the NC group, the survival rates of HK-2 in the Tf, AGE-Tf (33.3 mM), and AGE-Tf (500 mM) groups were significantly decreased (*p* < 0.05). The survival rates of HK-2 in the AGE-Tf (500 mM) and AGE-Tf (33.3 mM) groups were lower than that in the Tf group. Meanwhile, the survival rate of HK-2 in the AGE-Tf (500 mM) group was the lowest (**Figure 3A**).

Using AGE-Tf (500 mM) as the observation group, glucose concentrations were diluted to 50 mM, 100 mM, 200 mM, and 400 mM to stimulate HK-2 cells. Changes in HK-2 cell viability under different concentrations and time points were then observed. The results showed that HK-2 cell viability gradually decreased with increasing AGE-Tf (500 mM) concentration; however, there was no significant change in cell viability 24 h and 48 h after stimulation. After 72 h of stimulation, cell viability decreased significantly, as shown in **Figure 3B**.

### 3.4 Effect of AGE-Tf on apoptosis of HK-2 cells

**Figure 4:**
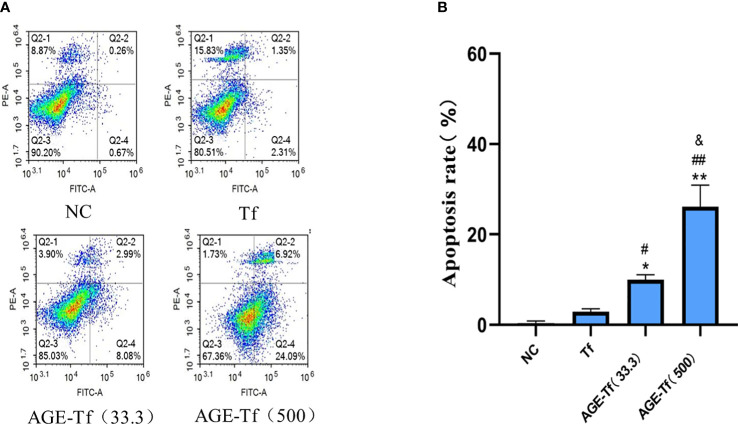
Effect of AGE-Tf on apoptosis of HK-2 cells. **(A)** HK-2 cell apoptosis diagram under different intervention conditions. **(B)** Statistical analysis of apoptosis rate. Compared with the NC group, *p < 0.01, **p < 0.001. Compared with the Tf group, *^#^p* < 0.05, *^##^p* < 0.01. Compared with the AGE-Tf (33.3) group, *^&^p* < 0.05. AGE-Tf (33.3): the glucose concentration was 33.3 mM when preparing AGE-Tf; AGE-Tf (500): the glucose concentration was 500 mM when preparing AGE-Tf.

The apoptosis rate of HK-2 cells stimulated by Tf, AGE-Tf (33.3 mM), and AGE-Tf (500 mM) for 72 h was determined by flow cytometry. The results showed that the apoptosis rates of the AGE-Tf (33.3 mM) and AGE-Tf (500 mM) groups [(9.96 ± 1.11) and (26.19 ± 4.73)] were significantly higher than that of the Tf group (2.90 ± 0.66) (*p* < 0.01). The apoptosis rate of the AGE-Tf (500 mM) group was higher than that of the AGE-Tf (33.3 mM) group (*p* < 0.05) (**Figures 4A, B**).

### 3.5 Effect of AGE-Tf on oxidative stress of HK-2 cells

**Figure 5:**
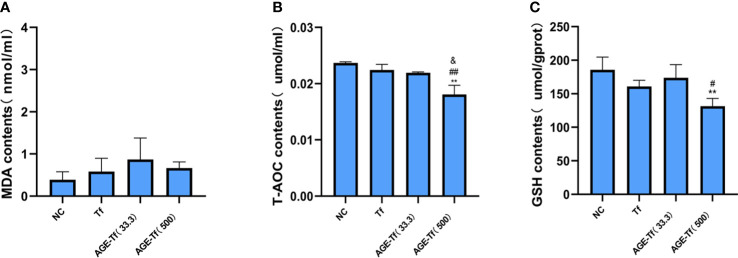
Effect of AGE-Tf on oxidative stress of HK-2 cells. **(A)** Changes in MDA level. **(B)** Changes in T-AOC level. **(C)** Changes in GSH level. Compared with the NC group, **p < 0.001. Compared with the Tf group, *^#^p* < 0.05, *^##^p* < 0.001. Compared with the AGE-Tf (33.3) group, ^&^ *p* < 0.05. AGE-Tf (33.3): the glucose concentration was 33.3 mM when preparing AGE-Tf; AGE-Tf (500): the glucose concentration was 500 mM when preparing AGE-Tf.

Compared with the Tf group, MDA levels in the AGE-Tf (33.3 mM) and AGE-Tf (500 mM) groups were increased but not significantly (*p* > 0.05) (**Figure 5A**).

T-AOC levels in the AGE-Tf (500 mM) group was significantly lower than that in the Tf and AGE-Tf (33.3 mM) groups (both *p* < 0.001); however, no significant difference was found between the AGE-Tf (33.3 mM) and Tf groups (*p* > 0.05) (**Figure 5B**).

GSH levels in the AGE-Tf (500 mM) group was significantly lower than that in the Tf group (*p* < 0.05). It was also lower than that of the AGE-Tf (33.3 mM) group, but not significantly (*p* > 0.05) (**Figure 5C**).

### 3.6 Effect of AGE-Tf on TfR expression level of HK-2 cells

**Figure 6:**
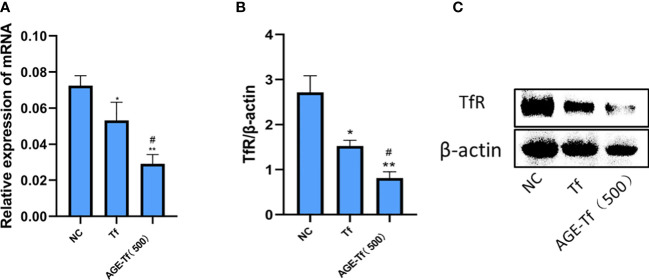
Effect of AGE-Tf on TfR expression level of HK-2 cells. **(A)** TfR mRNA analysis. **(B)** Gray-scale analysis of TfR protein expression. **(C)** TfR protein expression. Compared with the NC group, *p < 0.01, **p < 0.001. Compared with the Tf group, *^#^p* < 0.05; AGE-Tf (500): the glucose concentration was 500 mM when preparing AGE-Tf.

Compared with the NC group, the expression levels of mRNA in the Tf and AGE-Tf (500 mM) groups were significantly decreased (*p* < 0.05). Relating to the Tf group, TfR mRNA expression levels in the AGE-Tf (500 mM) group was significantly lower (*p* < 0.05) (**Figure 6A**).

Compared with the NC group, TfR protein expression levels in the Tf and AGE-Tf (500 mM) groups were significantly decreased (*p* < 0.05). Compared with the Tf group, the expression level of TfR protein in the AGE-Tf (500 mM) group was significantly decreased (*p* < 0.05) (**Figures 6B, C**).

## 4 Discussion

In this study, AGE-Tf was prepared by incubating Tf with different glucose concentrations (0 mM, 5.6 mM, 11.1 mM, 33.3 mM, 100 mM, 500 mM, and 1,000 mM) at 37°C for 14 days *in vitro* under sterile conditions. The results showed that the contents of fructosamine in each sugar-added group were higher than that in the Tf group. The increase was most significant when the glucose concentration was at 100 mM, 500 mM, and 1,000 mM (all *p* < 0.05), suggesting that Tf glycation degree gradually increased with increasing glucose concentrations *in vitro*.

Lactoferrin is a special form of Tf that exists in tears and colostrum. The levels of glycated lactoferrin in the tears of patients with diabetic retinopathy were also increased (21), and the degree of N-glycated lactoferrin in the milk of patients with gestational diabetes also increased significantly (22). Diabetic patients had an increased degree of non-enzymatic glycation of serum Tf (13, 23). Moreover, the degree of glycation increased from 1% to 2% in healthy individuals to 5% in adult diabetic patients (23), while it increased from 4% in healthy children to 11% in children with type 1 diabetes (24).

Tf glycation hinders its function as a high-affinity iron-binding protein (9). Understanding the effect of glycation on Tf function is the basis for a comprehensive understanding of the mechanism of iron homeostasis loss in diabetes mellitus. In this study, AGE-Tf TIBC levels decreased significantly with increasing glucose concentrations (33.3 mM, 100 mM, 500 mM, and 1,000 mM). Tf is prone to glycation in high glucose states, with the iron binding ability of glycation-modified Tf being significantly weaker than Tf. This may be due to the destruction of hydrogen bonds caused by glycation. Hydrogen bonds are key to stable iron binding, which blocks the acid–base mechanism of iron release in cells and seriously damages protein function (9). The holo-Tf glycation site is far from the iron binding site, and the abnormal iron release caused by glycation may be related to the cumulative effect of the destruction of hydrogen bonds within the holo-Tf molecule and instability of the overall structure caused by electrostatic interactions (10). Sadaki et al. (25) found that iron ions in unglycated Tf molecules loosely combine with proteins and have redox activity. Glycated holo-Tf generates oxygen radicals including O^2−^ and •OH through *in vivo* and *in vitro* experiments.

We identified the glycation sites and AGE modification types at each site of Tf and AGE-Tf (500 mM) by LC-MS/MS. Seventeen glycation sites in AGE-Tf (500 mM) and four glycation sites in Tf were detected. The AGE modification types in AGE-Tf (500 mM) were significantly elevated than those in the Tf group. Alena et al. (26) found that glycated sites of high-content plasma proteins were different between type 2 diabetic patients and healthy subjects of the same age and sex by LC-MS. They also found six glycated sites (Lys553, Lys659, Lys258, Lys299, Lys315, and Lys225) of plasma Tf in type 2 diabetic patients. Among them, the sites K225, K258, K553, and K659 were consistent with AGE-Tf (500 mM) glycation sites in the present study. Moreover, Golizeh et al. (8) found that compared to normal controls, six lysine residues (Lys103, Lys206, Lys276, Lys296, Lys534, and Lys640) and one arginine residue (Arg678) were modified *via* glycation in the serum Tf of type 2 diabetic patients, as assessed by shotgun proteomics and heavy water (^2^H_2_O)-based metabolic labeling method. Andrej et al. (27) reported that the plasma Amadori-peptide contents in patients with type 2 diabetes was significantly higher than that in healthy subjects. They also found that high glucose status had varying degrees of influence on the glycation site of human plasma proteins, and MS analysis found that glycation modification occurred at K683 site of Tf protein. Silva et al. (9) also found that Tf glycation sites increased significantly under hyperglycemia.

Due to the heterogeneity and instability of AGEs, the actual chemical structure of AGEs is not completely clear (28). It was reported that lysine-derived AGE modifications include Fructosyl-lysine (FL), Pyrraline (Pyr), Nϵ-Carboxymethyl-lysine (CML), and Nϵ-carboxyethyllysine (CEL) (1, 29–33). Arginine-derived AGE modifications include Argpyrimidine (ArgP), carboxymethyl arginine (CMA), Hydroimidazolone 1 (G-H1), Hydroimidazolone 1 (MG-H1), and the six-membered pyrimidine ring derivatives (1, 34). Similarly, the types of AGEs in glycation Tf identified by LC/MS-MS in this study include CML, G-H1, Fructosyl-lysine-1H2O, FL, and MG-H1.

AGE formation is accompanied by the production of ROS, which further react and damage proteins and other important biological molecules (18). As irreversible products of non-enzymatic reduction of sugars and amino groups of proteins, AGEs are metabolized and excreted by the kidney. However, if AGEs are not metabolized, they deposit in the kidneys and bind to AGE receptors (RAGE), which cause various pathological changes, including oxidative stress, apoptosis, and inflammation (35). The AGE and RAGE axis play a role in diabetic nephropathy (36, 37). In the present study, adding AGE-Tf (33.3 mM) and AGE-Tf (500 mM) to stimulate HK-2 *in vitro* showed that glycated-Tf promoted apoptosis of HK-2 cells and weakened its antioxidant capacity.

This study found that the expression levels of TfR in the AGE-Tf (500 mM) group were lower than those in the Tf group; however, the effect of Tf glycation on its receptor expression has not been reported yet; Tf glycation may hinder its binding to TfR. X-ray crystallography and cryoelectron microscopy mapped the Tf c-lobe region that interacts with TfR to the amino acid sequence residues 349–378 of Tf, and the residues K354, K365, and K380 located at or near these regions. When glycated, these undergo conformational changes, destroying the binding between Tf and TfR (10).

Moreover, CML, a member of the AGE family, disrupts cholesterol metabolism in HK-2 cells by activating sterol regulatory element binding protein 2 and liver X receptor. Afterwards, increased cholesterol synthesis mediated by 3-hydroxy-3-methylglutaryl-coA reductase, cholesterol intake mediated by LDL receptors, and cholesterol outflow mediated by decreased ATP-binding box transporter A1 ultimately lead to lipid accumulation in HK-2 (31). AGE associated with hyperglycemia was shown to increase tubular epithelial–myofibroblast trans-differentiation (TEMT) and extracellular matrix synthesis, leading to renal fibrosis (38). It was also proposed that autophagy protects renal TECs from injury in diabetic nephropathy. Liu et al. found that lysosomal membrane permeability and lysosomal dysfunction were triggered by AGEs, and the latter induced autophagy inactivation of renal TECs in patients with diabetic nephropathy (39).

However, the effect of Tf glycation on HK-2 cells has not been reported. Abnormal glycation of human immunoglobulin A (IgA) is associated with autoimmune disease pathogenesis, such as IgA nephropathy and IgA nephritic vasculitis (40). The apoptosis of human mesangial cells (HMCs) and HK-2 cells was accelerated, MDA levels were increased, SOD and GSH were decreased, and mRNA expression levels of RAGE and NF-κB genes were upregulated after AGE-BSA treatment *in vitro* (41). Moreover, exposure of HK-2 cells to 100 μg/ml AGE-BSA increased the production of ROS by 5.2 times and reduced GSH level (42). Lin et al. (43) investigated the role of core fucosylation on TGF-β receptor I and II (ALK5 and TGF-βRII) in the EMT of HK-2 cells. They found that inhibiting core fucosylation attenuated EMT in HK-2 cells despite high TGF-β1, TGF-βRII, and ALK5 levels. In addition, histone demethylase JMJD1A/Nuclear receptor subfamily 4 Group A member 1 (NR4A1) signaling may regulate the progression of renal tubular epithelial interstitial fibrosis induced by AGEs in HK-2 (44).

Studies have found that continuous exposure of HK-2 to AGE-BSA may damage mitochondrial function, elevate ROS production, and increase apoptosis of HK-2 cells (45, 46). Feng et al. (47) found that AGE-BSA increased the expression of ROS and monocyte chemotactic protein-1 (MCP-1) in renal mesangial cells by a time- and dose-dependent manner *in vitro*. The upregulation of ROS and MCP-1 levels induced by AGE-BSA was significantly blocked by RAGE neutralizing antibodies, which may have a potential impact on the pathogenesis of diabetic nephropathy. In addition, AGE and RAGE binding induces oxidative stress and chronic inflammation in renal tissue, involving the activation of NFκB, PI3K/Akt, and MAPK/ERK signaling pathways. These contribute to the progression of kidney disease (48–50). This study preliminarily confirmed that glycated Tf has a certain effect on apoptosis and antioxidant capacity of HK-2 cells; however, the exact mechanism needs further verification in the future.

## 5 Summary

The degree and sites of Tf glycation were increased *in vitro* induced by high glucose, while the binding ability of Tf to iron decreased. After HK-2 was stimulated by AGE-Tf *in vitro*, the apoptosis of cells was increased, the antioxidant capacity decreased, and the expression of TfR was downregulated.

## Data availability statement

The raw data supporting the conclusions of this article will be made available by the authors, without undue reservation.

## Author contributions

JL and YM conceived and designed the research. YM and QZ analyzed the data. PZ, XL, CG, and JG collected the data. YM and QZ wrote and revised the initial manuscript. JL supervised the whole study and revised the manuscript. All authors contributed to the article and approved the submitted version.
